# Utility of Weight-Bearing Computed Tomography in the Postoperative Assessment of Ankle Fractures

**DOI:** 10.3390/diagnostics15060750

**Published:** 2025-03-17

**Authors:** Mateusz Malik, Jakub Kwiatkowski, Artur Gądek, Agnieszka Lechowska-Liszka, Henryk Liszka

**Affiliations:** 1 Department of Orthopedics and Traumatology, University Hospital in Cracow, 30-688 Cracow, Poland; malik.mateush@gmail.com (M.M.); jakwiatkowski@su.krakow.pl (J.K.); artur.gadek@uj.edu.pl (A.G.); 2 Department of Orthopedics and Physiotherapy, Jagiellonian University Medical College, 30-688 Cracow, Poland; 3 Institute of Applied Sciences, Faculty of Motor Rehabilitation, University of Physical Education in Krakow, 31-571 Cracow, Poland; agnieszka.liszka@awf.krakow.pl

**Keywords:** ankle fractures, X-ray computed tomography, cone beam CT, lower-limb imaging

## Abstract

**Background:** Ankle fractures are among the most common injuries requiring surgical intervention. Standard radiographs are typically used for postoperative assessment; however, some patients continue to experience residual symptoms despite satisfactory radiographic outcomes. Weight-bearing computed tomography (WBCT), though not yet widely integrated into clinical practice, offers potential advantages in evaluating lower-limb deformities, injuries, and arthritis. This study explores the utility of WBCT for the midterm assessment following ankle fracture fixation and compares its findings with those obtained from standard radiographs. **Methods:** In this retrospective case study, we analyzed the correlations between the functional outcome scores approximately one year post-surgery and parameters assessed using WBCT. Pearson’s correlation coefficient was used to evaluate these correlations, and a *t*-test was performed to assess their statistical significance, with a threshold *p*-value of 0.05. Additionally, Spearman’s rank correlation coefficient was calculated as a supplementary descriptive measure, without significance testing. These correlations were then compared with those obtained from standard ankle radiographic views (anteroposterior, lateral, and mortise). **Results:** Several correlations were identified between WBCT parameters and functional scales, with certain parameters demonstrating high statistical significance (*p* < 0.05). Overall, the correlations observed for WBCT were stronger than those for standard radiographs. **Conclusions:** Although the study cohort was limited, the findings suggest that WBCT may provide additional insights beyond conventional radiography. Further research with larger patient groups is needed to establish its clinical relevance.

## 1. Introduction

### 1.1. Differentiating Weight-Bearing CT (WBCT) from Conventional CT

Weight-bearing CT (WBCT) remains a relatively niche modality in diagnostic imaging across most regions, although its technology is not novel, and it has garnered increased attention over the past decade. Its principles diverge significantly from those of conventional CT.

The primary distinction lies in the upright standing position afforded to the patient during WBCT, enabling weight-bearing conditions, whereas classical CT involves a supine positioning, with the patient passing through a detecting tube. This upright stance during imaging yields fundamentally different images, wherein bone alignment varies slightly, offering a more precise visualization of weight-bearing-related disturbances [1,2,3,4,5,6,7,8].

Additionally, WBCT typically delivers a lower radiation dose to the patient, attributable to its utilization of a three-dimensional conical beam instead of the standard fan-shaped beam [5,9]. Moreover, a single rotation of WBCT can capture more images compared to classical CT, which requires multiple rotations to obtain an equivalent image volume.

Presently, WBCT adoption remains limited due to factors such as restricted availability, insufficient familiarity among diagnostic practitioners, and marginally higher costs relative to radiography [1,10,11,12]. However, an increasing body of literature demonstrates emerging applications of WBCT in clinical practice [1,6,7,8,13,14,15,16,17,18,19,20,21,22,23,24].

### 1.2. Potential Limitations in Assessing Results Using Conventional Radiography

Ankle fractures are among the most prevalent injuries requiring surgical intervention, encompassing a spectrum of subtypes ranging from simple lateral malleolus fractures to complex trimalleolar injuries, particularly when accompanied by syndesmotic injury [25,26,27,28,29]. Postoperative assessment predominantly entails a series of radiographs; however, only a handful of parameters are considered reliable for measurement [30,31,32,33]. Despite favorable radiological outcomes, functional recovery may be suboptimal [1,34,35], thus rendering reliance solely on radiographs questionable.

Ensuring adherence to meticulous criteria during examination is paramount to yield precise and credible results, a task compounded by the inherent poor reproducibility of the parameters in radiographs. As elucidated by Spiros G. Pneumaticos’ study [32], most parameters exhibit diametrical changes with external and internal rotation, barring the width of the tibiofibular clear space, which remains relatively invariant. Consequently, even minor deviations can significantly impact the measurements, compounded by the challenge of discerning subtle positioning errors from radiographic images. Notably, comparing measurements between limbs introduces an additional source of potential error, since rotational misalignment may occur independently for each limb.

Recent research has underscored the tibiofibular clear space as the most reliable parameter for diagnosing syndesmosis in anteroposterior views [32], contrary to prior emphasis on its utility in mortise views [33], prompting its adoption in subsequent studies. Conversely, parameters such as the tibiofibular overlap (TFO) and medial clear space (MCS) are susceptible to considerable variation with malrotation, rendering them less reliable indicators of the syndesmosis integrity [30,32]. Therefore, the quest for more precise measurement tools persists in enhancing the diagnostic accuracy and guiding therapeutic interventions.

### 1.3. Approaches to Potential Challenges

Introducing three-dimensional examinations akin to classical CT imaging appears to address the technical limitations associated with radiographs, yet logistical challenges for patients may arise. While bilateral examinations are not commonly undertaken outside of research settings, unilateral scans often lack sufficient data to adequately distinguish syndesmotic pathologies from anatomical variations. Although ordering bilateral scans is feasible, expanding radiography diagnostics is seldom practiced; unilateral exams are typically favored. However, this approach can incur additional costs and radiation exposure for the patient [12]. Moreover, the need for separate measurements from two distinct exams is inconvenient for both the patient and the clinician. Furthermore, prolonged access times to computed tomography facilities pose significant challenges in numerous healthcare settings worldwide [36,37].

In our clinic, weight-bearing CT (WBCT) is emerging as a promising alternative for post-fixation ankle assessment. This modality combines the weight-bearing positioning capability of conventional radiographs with the three-dimensional examination afforded by classical CT scans. Moreover, the bilaterality of WBCT facilitates easier comparison with the unaffected side. Importantly, patient access times for WBCT exams are notably shorter, particularly in our clinic, where the machine is primarily dedicated to orthopedic patients. Consequently, WBCT is emerging as the preferred imaging modality for our patients, often replacing classical CT scans. Standard radiographs are also conducted for comparative purposes.

### 1.4. Aim of the Study

Our study focused on the midterm assessment following ankle fracture fixation using both WBCT and plain radiographs. The objective of this study was to investigate potential correlations between the parameters measured in WBCT scans and the functional outcomes of the patients. Our null hypothesis posited that there is no association between the results of the WBCT measurements and the functional scales assessed one year postoperatively. Subsequently, we compared these findings with the correlations observed between the functional scales and radiograph measurements.

## 2. Materials and Methods

### 2.1. Overall Description

#### 2.1.1. Inclusion and Exclusion Criteria

In this retrospective case study, we included 30 patients (12 males, 18 females; mean age: 51.77 years, range: 25–74 years) who underwent ankle fracture fixation surgery at our clinic between 2020 and 2021 and had undergone computed tomography scans using the weight-bearing CT machine around 1 year post-op, as well as standing bilateral radiographs. Subgroups were not delineated for this specific study, and the cohort comprised patients with isolated unilateral Weber B and C fractures.

The exclusion criteria included the following: Weber A fractures, pilon fractures, bilateral fractures, pelvic fractures, additional or previous fractures in the lower limbs, polytrauma, severe arthritis of the lower-limb joints, previous surgeries on the lower limbs, history of vascular disease (e.g., venous thrombosis), peripheral neuropathy, and prior ligamentous injuries to the lower limbs.

#### 2.1.2. Institutional Review Board Statement

Our Institutional Review Board approved the study.

#### 2.1.3. Postoperative Examination and Clinical Assessment

On average, at one-year post-surgery, all patients underwent weight-bearing CT and bilateral standing radiographs. Additionally, clinical examinations were conducted during routine visits to our clinic, encompassing data collection from functional scale questionnaires, including the Visual Analogue Scale for Hindfoot and Ankle (VAS-HA), European Foot and Ankle Society (EFAS) Score, American Orthopedic Foot and Ankle Society (AOFAS) Score, Olerud–Molander Ankle Score (OMAS), and Foot and Ankle Ability Measure (FAAM). These tools are recognized for their efficacy in assessing postoperative outcomes and function [38,39,40,41,42,43,44,45,46,47,48,49,50,51].

#### 2.1.4. Equipment Utilized in Radiological Examination

For CT scans, a weight-bearing CT LineUp^TM^ machine (CurveBeam AI, 2800 Bronze Dr. Suite 110, Hatfield, PA, USA 19440) was used with the following exposure settings: 720 frames, 120 kVp, 5.0 mA, 12 ms. For radiographs, a Siemens Ysio Max machine (Siemens Healthineers AG, Siemensstr. 3, 91301 Forchheim, Germany) was used with the following exposure settings: 52 kV, 5 mAs.

#### 2.1.5. Software Used for Measurements

Parameters were evaluated using the CubeVue 3.7.0.3 software (CurveBeam AI, 2800 Bronze Dr. Suite 110, Hatfield, PA, USA 19440) for WBCT analysis and AGFA IMPAX 6.7.0.6011 (AGFA Healthcare, Septestraat 27, 2640 Mortsel, Belgium) for the evaluation of radiographs, with a measurement accuracy of 0.1 mm for distances and 0.1° for angles. CubeVue is a specialized software designed for processing weight-bearing CT images, providing advanced features such as 3D reconstructions, multiplanar reformats, and precise measurement tools for assessing joint alignment, bone morphology, and fractures. The program enables a detailed evaluation of the ankle joint in its natural weight-bearing position, which is essential for accurately assessing fractures and deformities that might not be visible in traditional, non-weight-bearing CT scans. AGFA IMPAX 6.7.0.6011, on the other hand, was used for analyzing radiographs, offering precise and reliable measurements for standard two-dimensional imaging of the ankle joint.

#### 2.1.6. Parameters Selected for the Study

For WBCT analysis, parameters focusing on tibial incisura anatomy [24,25,26,50,51,52,53,54,55] were selected:

1. Depth of the incisura (depth);
2. Engagement of the fibula within the incisura (engagement);
3. Rotation of the incisura (rotation);
4. Protrusion of the fibula within the incisura (protrusion);
5. Torsion of the fibula (torsion).

Further description is provided in Section 2.2.

For the radiograph assessments, parameters commonly used for ankle evaluation were employed:

1. Ankle joint space;
2. Medial clear space;
3. Lateral clear space;
4. Fibular overlap;
5. Syndesmosis width (mortise view).

Further description is provided in Section 2.3.

#### 2.1.7. Statistical Methods

First, we used a Shapiro–Wilk test to assess the normality of our data. To evaluate the correlation between the measured parameters and functional scores, we applied Pearson’s correlation coefficient for each parameter. The significance of the correlation was determined using a *t*-test. Additionally, we calculated Spearman’s rank correlation coefficient as a supplementary measure to assess broader relationships, though without testing for significance.

Pearson’s correlation coefficient was chosen as the primary method for evaluating the strength and direction of linear relationships between normally distributed continuous variables. This method was specifically used to analyze correlations between functional outcomes (measured on interval scales) and quantitative parameters from WBCT and radiographs, with significance determined by a *p*-value threshold set at 0.05.

In contrast, Spearman’s rank correlation coefficient was used to assess monotonic relationships, especially when the data did not meet the normality assumption or when ordinal data were involved. Unlike Pearson’s correlation, Spearman’s method does not require normally distributed data and is capable of detecting consistent monotonic trends.

By prioritizing Pearson’s correlation and supplementing it with Spearman’s correlation, we aimed to provide a comprehensive approach to understanding the relationships between the assessed parameters and functional outcomes.

#### 2.1.8. Calculations and Reliability

For each patient, three surgeons performed measurements of the parameters twice at three different levels for both ankles, resulting in a total of 18 values per parameter. The mean values from these measurements were then calculated and used for further analysis.

Differences between the injured and non-injured sides were computed, and absolute values were recorded for statistical evaluation.

Measurement reliability was assessed using the intra-class correlation coefficient (ICC) to determine both intra-rater and inter-rater reliability. The standard deviation (SD) for each parameter was also calculated.

Correlations between the measured parameters and functional scales were analyzed using Pearson’s and Spearman’s rank correlation coefficients. Statistical significance for Pearson’s correlation was determined based on the *p*-value. Statistical analyses were performed using Statistica 13.3 (SoftStat Polska Sp. z o.o., ul. Cystersów 9, 31-553 Cracow, Poland) and Microsoft Excel 2013 v15.0.5589.1000 (Microsoft Corporation, 1 Microsoft Way, Redmont, WA 98052, USA).

### 2.2. Detailed Description of WBCT Parameters

At present, standardized protocols for measuring parameters in the WBCT of ankles have not yet been established [1,56]. While numerous studies have proposed beneficial parameters for syndesmosis assessment that have been utilized in the research [21,57,58,59,60,61], our methodology opted for the parameters outlined in Boszczyk et al.’s 2017 article [26], assessing the tibial incisura anatomy in classical CT. We made slight modifications to the measurement acquisition method to facilitate obtaining data in bilateral examinations, as well as minor adjustments to the parameter names. The CubeVue softwere was utilized for the process, with all parameters studied in the axial view. Additionally, the coronal view was employed for controlling and adjusting the correct levels of the measurements.

**Figure 1:**
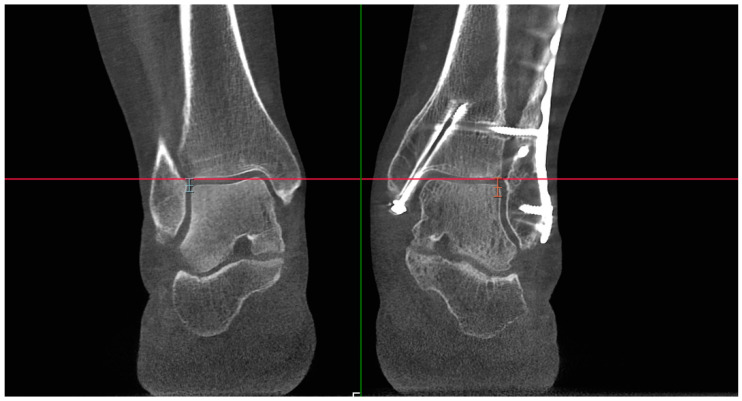
The transverse line (marked in red) is adjusted to intersect both ankles at equivalent levels, utilizing the lateral margins of the tibial plafonds as references. The green line represents the sagittal plane, which is irrelevant to the adjustment process. The two measurement lines visible in the figure (light-blue and orange lines) extend distally from the transverse line (red), indicating the lowest and highest levels for further measurements: 5 mm for the light-blue line and 7 mm for the orange line. Other levels of measurement are not marked in the figure.

First, a transverse line was positioned in the coronal plane to symmetrically intersect the lateral marginal point of the tibial plafonds in both ankles (Figure 1).

Adjusted in this manner, the line enables us to measure both ankles with a single cut. Based on our experience, this approach reduces the time required for measurements and improves the agreement between observers. Therefore, it has become our preferred method instead of adjusting separate planes for each ankle.

Second, the levels for further calculations were distinguished by perpendicularly measuring the distances proximal and distal to the transverse line. For parameters 1, 2, 3, and 4, measurements were conducted separately at various levels: 7, 8, and 9 mm proximal to the lateral margin of the tibial plafond; for parameter 5, measurements were taken at 5, 6, and 7 mm distal from the lateral margin of the tibial plafond (Figure 1).

**Figure 2:**
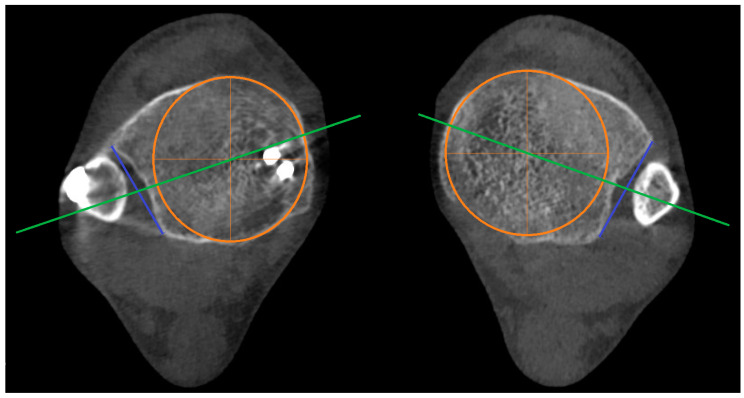
Reference Line A marked blue and reference Line B marked green, as described in the text. Orange color represents circular incision in the tibia. Line B connects the midpoint of Line A and the center of the circular incision in the tibia.

Before measurements were taken in axial views on separate levels, reference lines (Line A and Line B) were drawn. Line A links the anterior and posterior margins of the tibial incisura, while Line B connects the midpoint of Line A with the center of the circular incision in the tibia (Figure 2).

**Figure 3:**
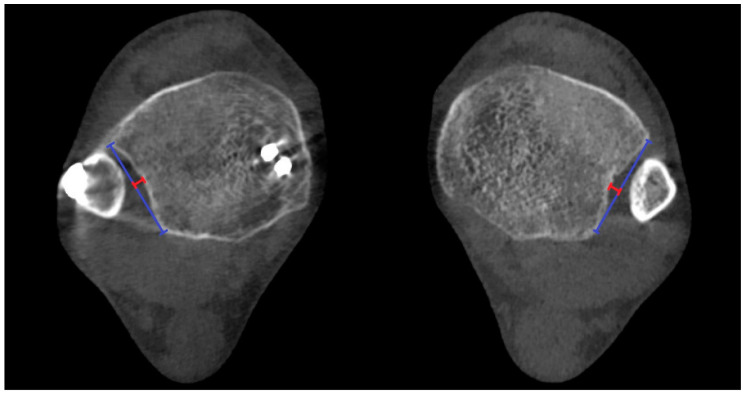
The red line represents the ‘depth’, and the blue line represents the reference Line A.

**Figure 4:**
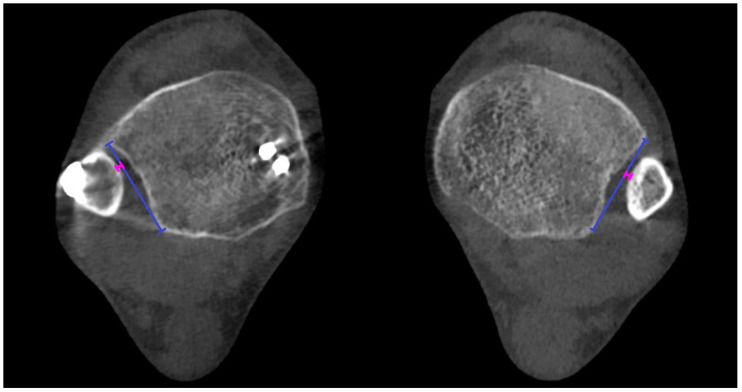
Bilateral presentation of variant 2a of ‘engagement’ shown on both sides with pink lines—the distance between reference Line A (marked blue) and the shortest distance along a perpendicular line to the cortex of the fibula.

**Figure 5:**
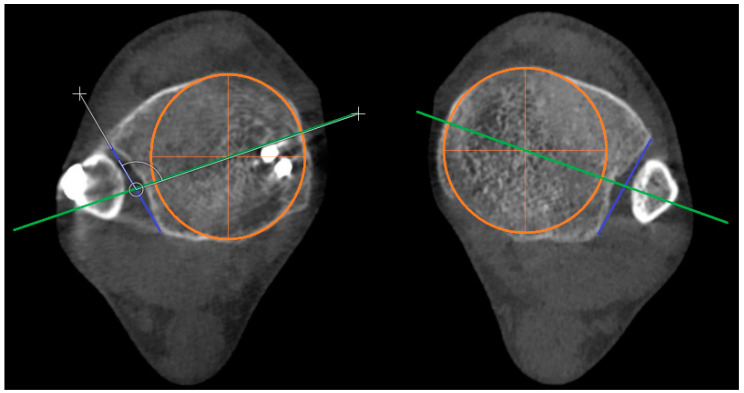
The angle is measured between reference Line A (marked blue) and reference Line B (marked green). The incisura angle is determined by subtracting 90° from the measured angle value. The circular incision in the tibia is marked in orange.

**Figure 6:**
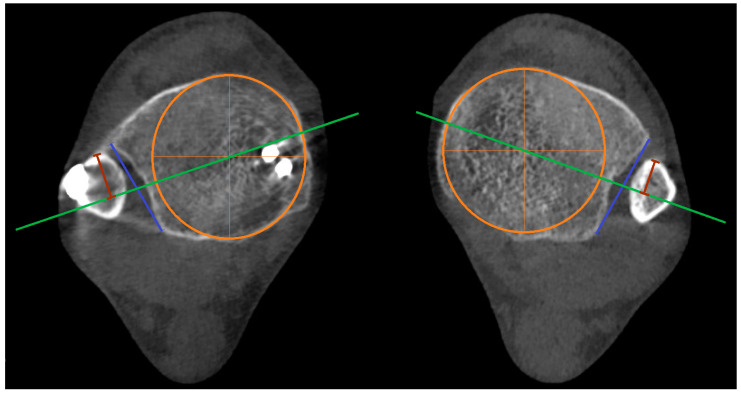
‘Protrusion’ is marked with a brown line. Reference lines are marked blue (Line A) and green (Line B). The circular incision in the tibia is marked in orange.

**Figure 7:**
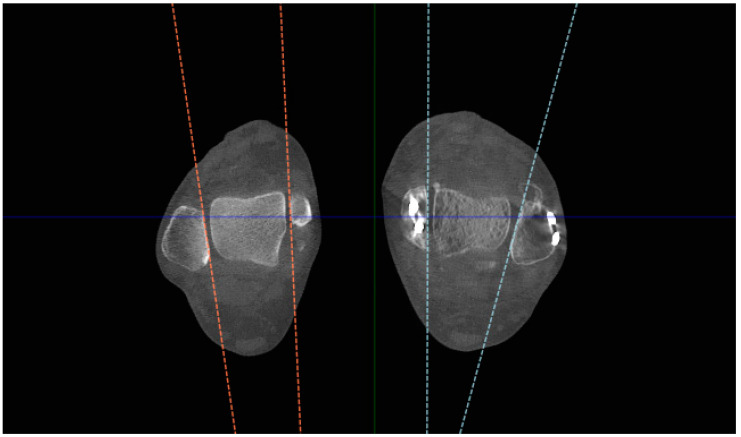
Torsion of the fibula (torsion) angles measured in both ankles. Measurements were taken in the axial plane, 5, 6, and 7 mm distal to the lateral margin of the tibial plafond. Orange lines represent the articular surfaces of the lateral and medial malleolus of the unaffected ankle, while light-blue lines correspond to the affected ankle. The angle of the unaffected ankle is measured between the orange lines, and the angle of the affected side is measured between the light-blue lines. Dark blue represents transverse line which is not relevant for the measurments.

1. Depth of the incisura (depth)—This parameter is utilized to assess the depth of the tibial incisura, defining the longest distance between referential Line A and the cortex of the incisura along a perpendicular line. ‘Depth’ is depicted as the red line in the figure below (Figure 3);
2. Engagement of fibula within incisura (engagement)—two variants of measurement are possible:
  2a. When the fibular cortex does not intersect Line A, the parameter describes the shortest distance possible to the fibular cortex along a perpendicular line, with values being negative (Figure 4);
  2b. When the fibular cortex intersects Line A, the parameter describes the longest distance possible in a perpendicular line to the fibular cortex in the direction toward the tibial bone, with values being positive;
3. Rotation of the incisura (rotation)—This parameter is determined by measuring the angle between referential Line A and Line B, subtracting 90 degrees (Figure 5). When Line A and Line B are perfectly perpendicular, the result is 0. A positive result indicates retroversion of the incisura, while a negative result indicates anteversion;
4. Protrusion of the fibula (protrusion)—This parameter assesses the anterior or posterior alignment of the fibula within the incisura. It is determined by measuring the longest possible distance in a perpendicular line between Line B (green) and the fibular cortex in the anterior direction. As depicted in the figure below, this is illustrated by the brown lines on both ankles (Figure 6);
5. Torsion of the fibula (torsion)—This parameter is employed to assess whether the fibula is malrotated. It simply measures the angle between the lateral malleolar facet and the medial malleolar facet in distal cuts (5, 6, 7 mm distal to lateral margin of tibial plafond). A positive value indicates external rotation of the fibula, while a negative value indicates internal rotation (Figure 7).

### 2.3. Detailed Description of Radiograph Parameters

All parameters listed below were evaluated bilaterally, with each parameter measured at three different levels (except for fibular overlap, which was assessed only at its widest point; instead, three measurements were taken). Each measurement was repeated by every investigator. AGFA IMPAX 6.7.0.6011 was utilized for calculating the measurements.

To ensure the highest possible reproducibility of the measurements and to minimize the risk of asymmetry due to potential rotational misalignment, all ankle radiographs were routinely calibrated. Given the nature of this study, single-point measurements would not have been appropriate, as they could either underestimate or overestimate the abnormalities.

Although the specific measurement method applied in this study has not been previously validated in other research, it was designed to accurately capture potential joint space narrowing observed in radiographic imaging. This approach ensures that any existing abnormalities are reflected in the measurements.

For example, when assessing the ankle joint space, early degenerative changes may lead to localized narrowing, such as medial joint collapse, while the lateral aspect remains unaffected. Single-point measurement in such cases could yield misleadingly normal results. To address this limitation, we conducted measurements at three distinct levels within the joint, reducing the likelihood of overlooking localized abnormalities. A similar approach was applied to the other parameters examined in this study.

For all joint space measurements (1, 2, 3), equal intervals were utilized to enhance the repeatability of the calculations. Three proportional lines were employed to establish reference points for measuring the joint space:

Line X: drawn from the tip of the medial malleolus to the curve connecting the medial malleolus with the tibial plafond;

Line Y: drawn from the tip of the fibula to the most medial point of the tibial plafond;

Line Z: established by connecting the highest points of the talar bone and extending the line to make contact with the fibular and tibial cortex within the joint.

**Figure 8:**
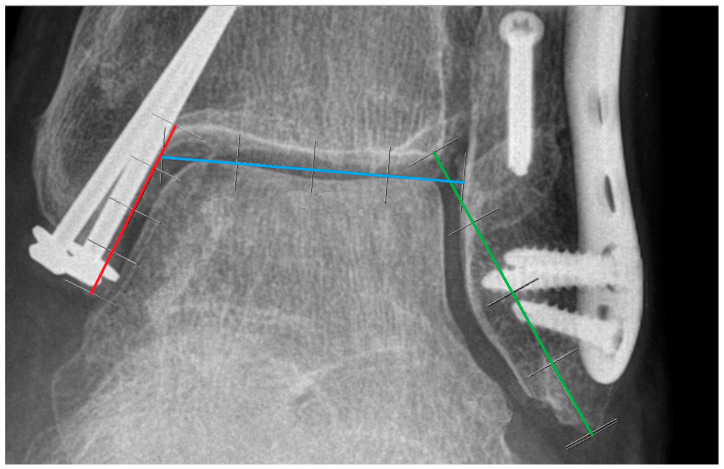
Three lines of proportion were used to establish reference points for measuring the joint space: Line X (red), Line Y (green), and Line Z (blue). All lines are divided into four equal parts. Line X is defined from the tip of the medial malleolus to the curve connecting the medial malleolus with the tibial plafond. Line Y is defined from the tip of the fibula to the most medial point of the tibial plafond. Line Z is established by connecting the highest points of the talar bone and extending the line to make contact with the fibular and tibial cortex within the joint.

Lines X, Y, and Z are presented below (Figure 8).

**Figure 9:**
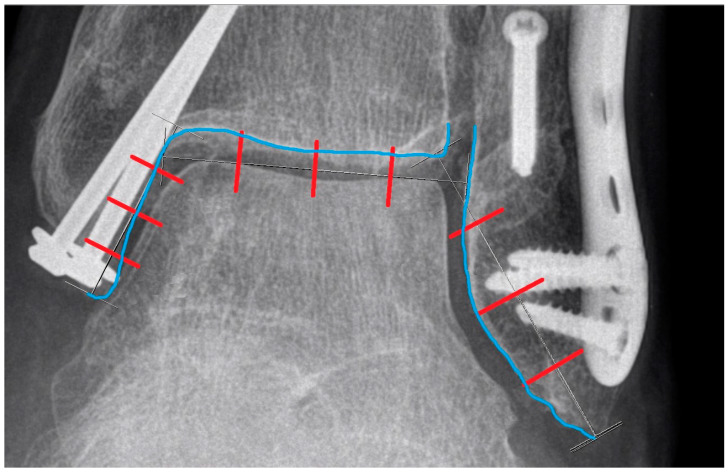
The blue line represents the cortical contour of the tibia and fibula. The red lines divide the previously shown Lines X, Y, and Z into four equal parts. These red lines are extended perpendicularly and intersect with the blue contour, creating reference points for further measurements.

Then, Lines X, Y, and Z were proportionally divided into four equal parts by three perpendicular lines, which were extended to intersect the contour of the tibial and fibular cortex, creating our points of reference (Figure 9).

**Figure 10:**
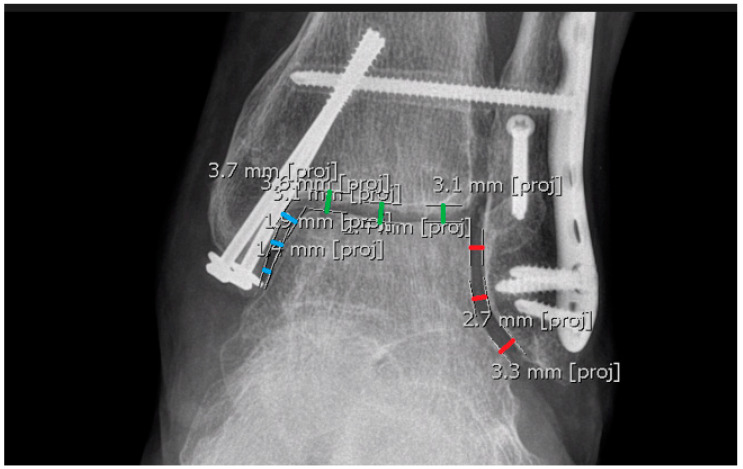
Measurements without reference lines. The red, green, and blue lines represent measurements for lateral clear space, ankle joint space, and medial clear space, respectively. The exact values displayed in the figure are not essential.

Measurements:

1. Ankle joint space—measurements taken from 3 different reference points on the tibial plafond (indicated in green in the figures below) (Figure 10);
2. Medial clear space—measurements taken from 3 different reference points on the medial malleolus (indicated in blue in the figures below) (Figure 10);
3. Lateral clear space—measurements taken from 3 different reference points on the lateral malleolus (indicated in red in the figures below) (Figure 10).

**Figure 11:**
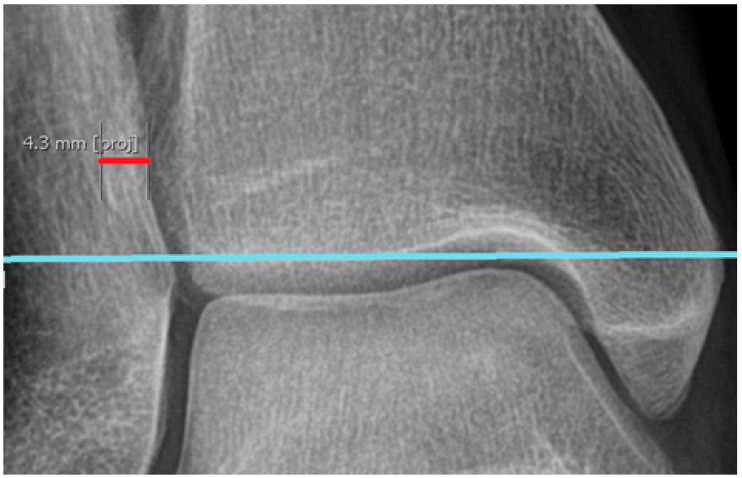
The red line represents the width of the fibular overlap, while the blue line indicates the plane of the tibial plafond. These lines are parallel.

**Figure 12:**
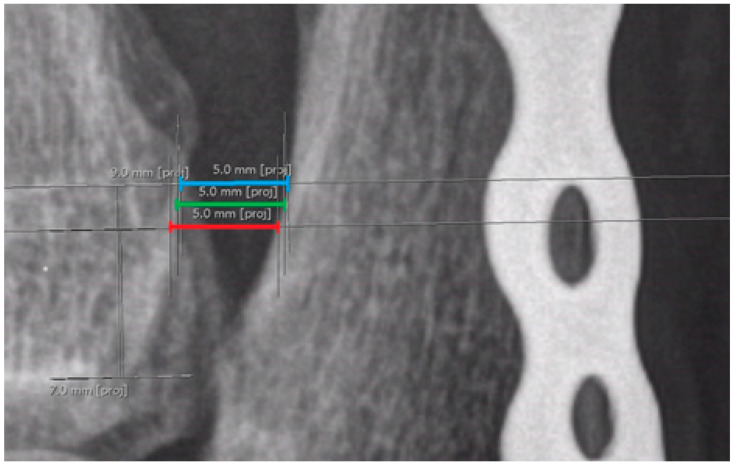
Three separate measurements of syndesmosis width in the mortise view, taken at three different levels: blue, green, and red lines represent measurements 9 mm, 8 mm, and 7 mm above the lateral margin of the tibial plafond, respectively.

When it was not possible to obtain one of the measurements of the lateral/medial clear space (i.e., proximal or distal), the other two measurements were used, supplemented by one additional measurement in between;

4. Fibular overlap—to measure the fibular overlap, we utilized 6 separate measurements (3 measurements taken twice at the widest point possible, along a line parallel to the tibial plafond) (Figure 11);
5. Syndesmosis width (mortise view)—to measure the syndesmosis width in the mortise view, we took measurements at 7, 8, and 9 mm above the tibial plafond. The measurements were parallel to the tibial plafond (Figure 12).

## 3. Results

Inter-rater and intra-rater reliability were assessed by calculating the intra-class correlation coefficients (ICCs) for measurements repeated by a single investigator and between the three investigators. The mean value of each parameter comprised a total of 18 separate measurements (3 surgeons, 3 different levels, 2 measurements). Standard deviations (SDs) were also calculated. The calculated ICCs for all combinations were above 0.9, confirming the reproducibility and reliability of our measurements. The intra-rater and inter-rater reliability for each parameter are presented in Table 1 and Table 2.

Pearson’s correlation coefficient and Spearman’s rank correlation coefficient were interpreted as positive when the value was above 0.3 (or below −0.3 for negative correlations). Values between 0.3 and 0.5 (or from −0.3 to −0.5) were considered as indicative of low positive (or negative) correlations, values between 0.5 and 0.7 (or from −0.5 to −0.7) were regarded as moderate positive (or negative) correlations, and values between 0.7 and 0.9 (or from −0.7 to −0.9) were considered indicative of high positive (or negative) correlations. Values below 0.3 (or above −0.3) were considered indicative of no correlation.

Among all the parameters measured in the WBCT, only a few demonstrated low correlations with the functional scales. No moderate or high correlations were identified. The remainder of the parameters exhibited no correlations whatsoever.

The most notable correlation was observed with ‘engagement’, which correlated with the VAS-HA (Table 3), EFAS Score (Table 4), and FAAM (Table 5), and showed a slight correlation with the Olerud–Molander Score (Table 5); correlations above 0.3 or under −0.3 were highlighted, as well as *p*-values under 0.05. However, the significance of the Pearson’s correlation coefficient (*p*-value) was under 0.05 only with the VAS-HA (*p* = 0.047) (Table 3) and FAAM (*p* = 0.01) (Table 5), although the *p*-value of the correlation with the EFAS Score was slightly above 0.05 (*p* = 0.085) (Table 4). Additionally, protrusion showed a correlation with the FAAM scale, with high significance at *p* < 0.05 (0.04). Compared to the parameters measured in the radiographs, those measured in the WBCT exams tended to have more significant values. Detailed calculated correlations are shown in Table 3, Table 4, Table 5, Table 6 and Table 7.

**Chart 1:**
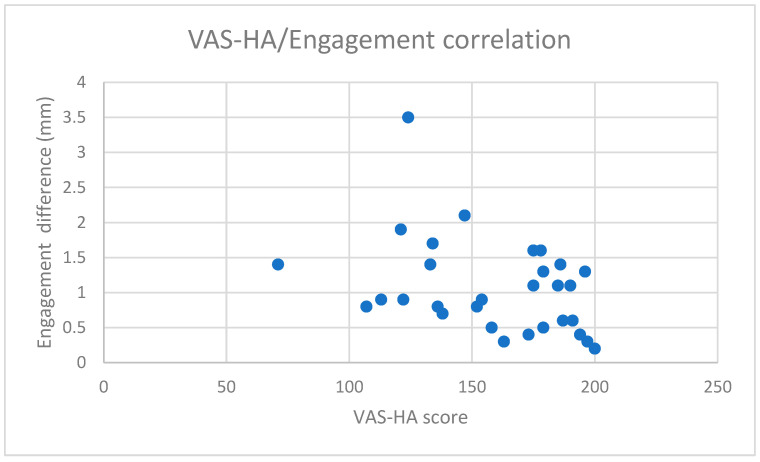
Correlation between VAS-HA and engagement difference in WBCT. The *X*-axis represents VAS-HA values (ranging from 0 to 200), while the *Y*-axis represents the absolute differences in ‘engagement’ between the affected and unaffected ankles (mm). The chart shows a low negative correlation (Pearson’s = −0.364; Spearman’s = −0.401) with high significance, as the *p*-value is below 0.05 (*p* = 0.047).

**Chart 2:**
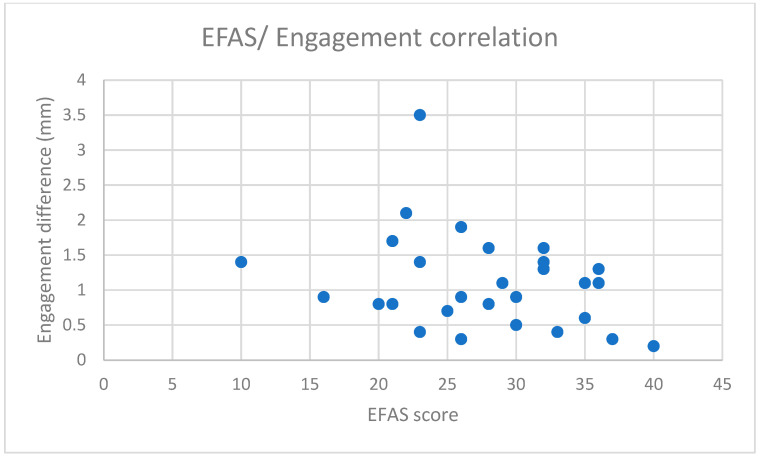
Correlation between the EFAS scale and engagement difference in WBCT. The *X*-axis represents EFAS Score results (ranging from 0 to 40), while the *Y*-axis represents the absolute differences in ‘engagement’ between the affected and unaffected ankles (mm). The chart shows a low negative correlation (Pearson’s = −0.318) with low significance, as the *p*-value is above 0.05 (*p* = 0.086).

**Chart 3:**
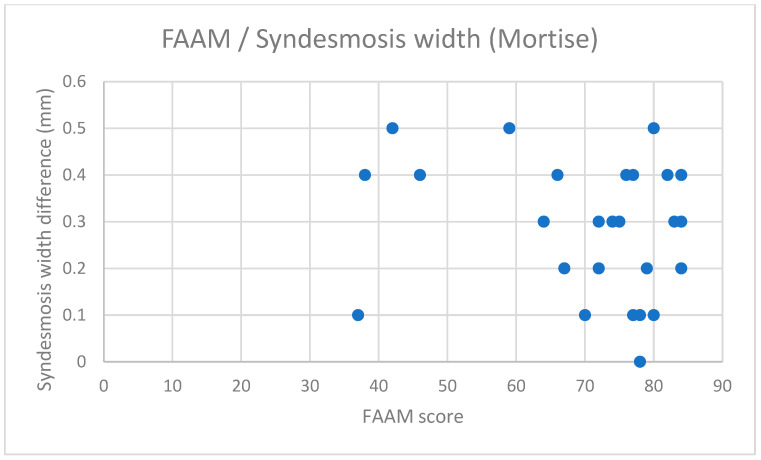
Correlation between the FAAM scale and syndesmosis width (mortise). The *X*-axis represents the FAAM Score results (ranging from 0 to 84), while the *Y*-axis represents the absolute differences in syndesmosis width in the mortise view (mm). This parameter was chosen as a representative radiographic measure that exhibited any correlation with functional scales. A low correlation is observed only in Spearman’s rank correlation coefficient (0.359), while Pearson’s correlation coefficient shows no correlation (−0.181). The correlation is not statistically significant, as the *p*-value is above 0.05 (*p* = 0.16). No evident correlation is visible on the chart, as the data points are more randomly distributed.

**Chart 4:**
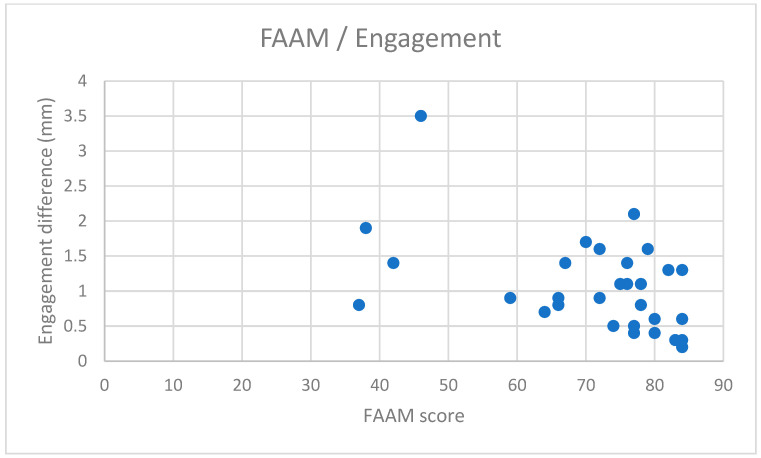
Correlation between the FAAM scale and engagement difference in WBCT. The *X*-axis represents the FAAM Score results (ranging from 0 to 84), while the *Y*-axis represents the absolute differences in ‘engagement’ between the affected and unaffected ankles (mm). The chart shows a low negative correlation (Pearson = −0.375) with high significance, as the *p*-value is below 0.05 (*p* = 0.01).

**Chart 5:**
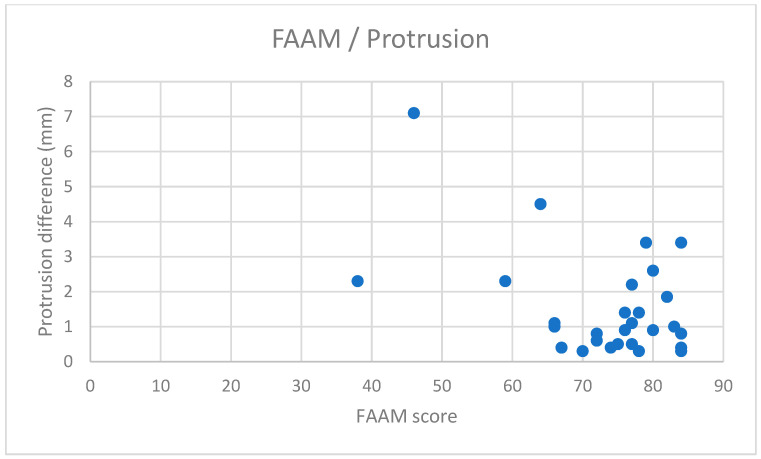
Correlation between the FAAM scale and protrusion difference in WBCT. The *X*-axis represents the FAAM Score results (ranging from 0 to 84), while the *Y*-axis represents the absolute differences in ‘protrusion’ between the affected and unaffected ankles. The chart shows a low negative correlation (Pearson = −0.375) with high significance, as the *p*-value is below 0.05 (*p* = 0.04).

The most significant correlations are presented in Chart 1, Chart 2, Chart 3, Chart 4 and Chart 5.

Within the functional scales, the AOFAS Score (Table 6) and the Olerud–Molander Score (Table 7) exhibited the least correlation with the measured parameters, each having only one parameter that barely correlated. Conversely, the FAAM Score (Table 5) emerged as the most correlating scale, with three parameters showing correlation.

## 4. Discussion

### 4.1. Is Weight Bearing Truly Necessary?

The necessity of weight bearing in imaging can be questioned. One study investigated whether patients exerted precisely 50% of their weight on the examined leg during standing weight-bearing radiographs [62]. The results raise questions about the reliability and reproducibility of equal weight bearing for both limbs, especially if there are differences in the measurements between them.

Once more, this study was limited to radiographs and involved a relatively small sample size. Enhancements in the results can be attained through subsequent trials of the examination, or potentially by providing clearer instructions to patients. Additionally, the validity of this study could be called into question due to the unclear importance of uneven weight distribution. In theory, variations in weight bearing—whether closer at 40% or 60%—should not substantially impact the parameter values in healthy ankles. Hence, the observed differences may not be as significant as anticipated.

Perhaps more studies should be conducted in the future to also address this issue for WBCT, whereas standing in a narrow tube could potentially create entirely different weight-bearing conditions compared to plain weight-bearing radiographs.

However, several factors support weight bearing in imaging. Most studies comparing weight-bearing and non-weight-bearing imaging from plain radiographs to MRIs demonstrate that the distribution of forces alters the alignment of the bones in relation to each other [8,24,61,63,64,65], completely changing the measurable parameters, which was the primary aim for conducting this study: to measure the parameters under patient-applied weight. Moreover, studies in which weight bearing is utilized can provide a more objective and precise approach for diagnosis [30]. Additionally, weight bearing, in some cases, can influence decision making before surgery, which is crucial for potential outcomes [66,67].

Also, further research on the necessity of weight bearing may be considered for future studies, such as comparing WBCT with classical CT, or maybe comparing WBCT with images obtained from the same cone beam machine taken in the sitting, non-weight bearing position.

To sum up, the literature indicates that weight-bearing exams seem to have more diagnostic value compared to non-weight-bearing exams.

### 4.2. Why Not Use Classical CT Instead of WBCT?

One might question the benefit of using WBCT when the same parameters could simply be assessed using classical non-weight-bearing CT scans. Few studies have been conducted on this specific topic, with many comparisons between weight-bearing and non-weight-bearing exams focusing on different parameters and involving small patient cohorts [8,61,68,69].

However, by utilizing conventional CT, we forego the diagnostic potential of weight bearing, as discussed in Section 4.1. While some form of substitution for weight bearing is feasible in some studies [70], it also necessitates a specially developed axial-loading device, which could pose challenges in recreating conditions akin to upright standing positions.

The parameters measured in classical bilateral CT scans when the axial load is not applied seemed insufficient in differentiating mild anatomic asymmetry from the malreduction of syndesmosis [71], although the results were not compared with WBCT.

Another factor in favor of WBCT is the lower radiation dose compared to classical CT scans [12,72], along with the reduced examination time for patients [12]. Additionally, WBCT may offer cost-effectiveness advantages in certain conditions [12].

Our study was conducted using WBCT instead of conventional CT due to the overall advantages it offers. However, further evaluation is warranted through future studies.

### 4.3. Is Radiography Alone Sufficient?

It is undeniable that plain radiographs remain the primary modality utilized in the diagnostics of the foot and ankle. They offer greater cost-effectiveness, availability, and significantly lower radiation doses compared to 3D examinations (both conventional CT and WBCT).

However, consideration must be given to the fact that the most commonly used radiographs (AP and lateral) can be insufficient; thus, some additional projections may provide more relevant data, as suggested by some studies [73,74], and can even be used intraoperatively [75]. Measurements obtained in radiographs may have poor reproducibility and, according to studies, often fail to correlate with findings from 3D imaging [31,76,77]. In our study, the correlation values were smaller compared to those obtained with WBCT (Table 3, Table 4, Table 5, Table 6 and Table 7), but many factors can be improved or changed for the continuation of our research.

Although in our study, two radiograph parameters showed some trace of correlation (‘syndesmosis width in mortise view’ correlated with the FAAM Score (Table 5, Chart 3) and lateral clear space correlated with the AOFAS Score (Table 6)), both had low significance, with ‘*p*-values’ above 0.05.

Furthermore, for the majority of the calculated values, no correlation was observed with the functional scales at all. This favors our exploration of WBCT as a more accurate tool for assessment.

We also should reevaluate the results with a larger study group.

### 4.4. Is a One-Sided Exam Reliable Compared to a Bilateral Assessment?

One of the primary concerns involves justifying the conduction of a radiation-based examination on a healthy ankle. However, comparing the affected ankle with the contralateral side provides valuable insights into potential anatomical disturbances. This approach helps discern whether the findings observed for the affected side indicate actual pathological changes or merely normal anatomical variants of the patient [78].

Classical CT and radiographic scans are typically conducted unilaterally in routine diagnosis, while the healthy side is rarely examined, and comparative studies are not commonly performed.

Therefore, conducting bilateral examinations could pose logistical challenges and increase patients’ radiation exposure. Some studies have solely focused on assessing the postoperative syndesmotic morphology using non-weight-bearing CT scans [57,59,79], conducting only bilateral examinations. Unilateral exams are primarily employed for preoperative assessment [80,81].

Consequently, by conducting a one-sided examination, we overlook a substantial amount of data crucial for assessment. In the context of our study, it is nearly impossible to demonstrate any correlation due to the anatomical variability among patients [26,52].

### 4.5. What About Perioperative Assessment?

The possibility of using WBCT in the early postoperative period after ankle fracture fixation is debatable due to weight-bearing restrictions, although some studies have attempted to identify patterns in which immediate weight bearing is safe [82]. Many studies suggest that early weight bearing after 2 weeks postoperatively does not lead to a higher complication rate compared to not bearing weight for 6 weeks [83,84]. Thus, the 2-week postoperative threshold may currently serve as a cut-off point for performing weight-bearing exams, but only a few studies differentiate between ‘protected’ and ‘as tolerated’ early weight bearing [85,86]. With certain patients’ restrictions and the presence of pain, achieving a consistent 50/50 percent weight distribution between both sides during the exam may be challenging [62].

More promising are studies assessing syndesmotic injuries and syndesmosis before surgery using WBCT [87,88]. For accurate perioperative assessment, better alternatives likely exist, such as 3D imaging, targeted 2D views, and ankle arthroscopy [75,89,90,91,92,93,94].

Perhaps investigating the utilization of the same cone beam machine with the patient in a seated position, thereby eliminating the need for applying weight, could be a potential avenue for future research. Our study primarily focused on the application of weight; hence, we did not explore the aforementioned method.

### 4.6. Are the Measurements Too Complex?

Another issue to consider is the complexity of manually collecting measurements, which can be challenging and time-consuming.

In our study, we aimed to recreate the results as accurately as possible; thus, each parameter was measured twice by three separate investigators on three different levels, resulting in 18 measurements per parameter for each patient. This gave us excellent agreement between our results (Table 1 and Table 2): the intra-rater and inter-rater agreements measured with the intra-class correlation coefficient in all cases were above 0.9.

Future directions include moving toward automated programs and artificial intelligence, which can aid in acquiring more reliable calculations [95,96,97,98,99,100], but these technologies are not yet widely implemented in diagnostic programs.

The absence of automated software in our study necessitated the manual acquisition of the measurements.

Despite initial challenges, our study yielded valuable data (Table 3, Table 4, Table 5, Table 6 and Table 7). We can now identify which parameters are most promising for further investigation. The most significant charts selected (Chart 1, Chart 2, Chart 3, Chart 4 and Chart 5) demonstrate some degree of linear correlation.

We should also consider measuring parameters utilized in other studies for our next steps of our research and compare them to our results [59,78,87,88,101].

### 4.7. Availability and Costs

WBCT is not commonly utilized, with only a few machines available in Poland. Some studies even exclude WBCT from the research process, as observed in meta-analyses studying the accuracy of imaging techniques in assessing syndesmosis [102]. Additionally, the cost factor varies; while some studies indicate cost-effectiveness [1,10,11,12], the actual expense primarily depends on the volume of exams performed. In cases where the number of examinations is low, the return on investment for the machine may be insufficient, leading to potential financial losses.

### 4.8. New and Similar Studies on the Topic

Weight-bearing CT is gaining attention in the examination of the lower limbs, yet standardized protocols for evaluating its results are still lacking [1,56]. Recent studies focusing on syndesmosis injury continue to provide more data each year, consistently indicating that WBCT is a valuable tool in diagnosing syndesmotic injuries. These studies employ various methods and parameters [87,101,103,104], with some even experimenting with the application of additional torque to enhance the results [105]. Despite variations in the study approaches, the general consensus remains that WBCT is effective in diagnosing syndesmotic injuries. As the availability increases, costs decrease, and new standardized protocols emerge, WBCT is likely to become even more popular in the future.

### 4.9. Limitations

These preliminary results are based on a small group of 30 patients, which may impact the statistical significance of the findings. A larger sample size is necessary to obtain more robust statistical data.

Our study did not categorize patients into subgroups, which may have influenced the statistical analysis and correlation levels. Future research should consider subdividing the patient population for a more comprehensive analysis.

The observation period in our study lasted approximately one-year post-surgery. Longer follow-up observations are required, as the degree of arthritis may worsen over time, potentially affecting functional outcomes.

For the sake of simplicity in the calculations, some available parameters were not utilized in our study. However, including additional parameters could influence the final results, and future research should consider comparing a broader range of parameters.

Our study focused on the most common radiograph projections, excluding potentially useful planes. Including additional radiograph views may warrant further investigation in future research.

Manual measurement acquisition may introduce variability compared to automated programs. Despite efforts to ensure reliability, factors such as variations in the radiograph positioning and quality, as well as human error from both technicians and patients, may have influenced the accuracy of the measurements. Additionally, the reliability between separate radiographs was not assessed in our study, highlighting a potential area for improvement in future research.

## 5. Conclusions

This study explored the relationship between the functional outcomes and radiological parameters assessed using both weight-bearing CT (WBCT) and standard radiographs in patients approximately one year after ankle fracture fixation. Despite the relatively small sample size of 30 patients, our preliminary findings suggest that the WBCT parameters exhibit a stronger correlation with the functional outcomes compared to standard radiographs, highlighting the potential clinical relevance of three-dimensional weight-bearing imaging in postoperative assessment.

The null hypothesis, which assumed no correlation between the functional scales and radiological parameters, was rejected based on our findings. Instead, our results support the alternative hypothesis, indicating that specific WBCT-derived measurements demonstrate statistically significant correlations with the functional outcomes. While this suggests that WBCT could be a valuable tool in midterm postoperative evaluation, further studies with larger cohorts are needed to validate these findings and refine the interpretation of specific parameters.

Importantly, our study was not designed to compare weight-bearing with non-weight-bearing imaging or to determine whether weight-bearing assessments inherently provide superior results. Rather, it aimed to investigate the association between imaging parameters and functional status in a clinically relevant, weight-bearing setting. Nevertheless, our results indicate that standard radiographs exhibited considerably weaker correlations with functional scales than WBCT, suggesting that WBCT may offer a more precise and informative evaluation of joint integrity and alignment following ankle fracture fixation.

These findings open up significant opportunities for further research in this field. A crucial next step would be to repeat the study on a larger patient cohort to enhance statistical power and improve the generalizability of the results. Additionally, conducting subgroup analyses could provide insights into whether specific fracture patterns or treatment methods influence the correlation between the radiological parameters and functional outcomes. Another important aspect would be the long-term follow-up of patients to determine whether the observed correlations remain stable over time and whether WBCT parameters can serve as predictive markers for long-term clinical outcomes.

Future research should also explore additional radiological parameters used in other studies to refine the understanding of their clinical relevance. Moreover, assessing the clinical significance of imaging findings in relation to real-world functional impairment could help bridge the gap between radiological assessments and patient-reported outcomes. Comparisons with conventional 3D imaging techniques such as CT and MRI may further clarify the advantages and limitations of WBCT in postoperative evaluation.

Another potential area of investigation is the impact of weight bearing on imaging results. Performing WBCT scans under both standing and seated conditions could help determine whether weight application significantly alters measurement outcomes. Additionally, it would be valuable to assess the reliability of standard radiographs by evaluating whether repeated imaging yields consistent results, thereby determining the reproducibility and accuracy of conventional radiographic assessments in postoperative monitoring.

By addressing these aspects, future studies could further establish the role of WBCT in postoperative evaluation, optimize measurement techniques, and provide valuable insights into its clinical applications.

## Figures and Tables

**Table 1:** Intra-rater reliability measured using intra-class correlation coefficients (ICCs) for three observers. All results are above 0.9, indicating excellent reliability for the parameters measured by each observer.

Parameters—WBCT	ICC (Observer 1)	ICC (Observer 2)	ICC (Observer 3)
Depth	0.96	0.94	0.93
Engagement	0.93	0.96	0.91
Rotation	0.91	0.92	0.91
Protrusion	0.94	0.93	0.92
Torsion	0.92	0.92	0.91
**Parameters—Radiographs**			
Ankle Joint Space	0.97	0.96	0.95
Lateral Clear Space	0.92	0.93	0.91
Medial Clear Space	0.94	0.93	0.93
Fibular Overlap	0.93	0.92	0.91
Syndesmosis Width (Mortise)	0.92	0.94	0.93

**Table 2:** Inter-rater reliability measured using intra-class correlation coefficients (ICCs) among the three observers. All results are above 0.9, indicating excellent reliability for the parameters measured between observers.

Parameters—WBCT	ICC
Depth	0.94
Engagement	0.92
Rotation	0.91
Protrusion	0.93
Torsion	0.92
**Parameters—Radiographs**	
Ankle Joint Space	0.96
Lateral Clear Space	0.91
Medial Clear Space	0.93
Fibular Overlap	0.92
Syndesmosis Width (Mortise)	0.93

**Table 3:** Correlations between measured parameters and the VAS-HA Score. The first column lists the parameters calculated from patient examinations, the second and third columns show the correlation coefficients (Pearson’s and Spearman’s rank), and the fourth column presents the significance of values calculated for Pearson’s correlation coefficient using a *t*-test (*p*-value). The correlation of ‘engagement’ with the VAS-HA Score is highlighted. A low correlation was observed for both Pearson’s correlation coefficient (negative correlation = −0.364) and Spearman’s rank correlation coefficient (positive correlation = 0.401), with *p*-values below 0.05 (*p* = 0.047). The remaining values indicate no correlation.

VAS-HA Correlation			
WBCT Parameter	Pearson	Spearman’s	*p*-Value
Depth	−0.1477102	−0.0083426	0.43556837
Engagement	**−0.3644136**	**0.40122358**	**0.04708882**
Rotation	0.09818077	−0.0804227	0.60547187
Protrusion	−0.241647	0.27230256	0.19756955
Torsion	−0.2158447	0.26362625	0.2513249
Radiograph Parameter			
Ankle Joint Space	−0.0830054	0.09888765	0.66255783
Lateral Clear Space	−0.1838207	0.21279199	0.3303087
Medial Clear Space	0.03794588	0.02847608	0.84210519
Fibular Overlap	−0.0595995	0.21212458	0.75424124
Syndesmosis Width (Mortise)	−0.1323589	0.24276974	0.48527109

**Table 4:** Correlations between measured parameters and the EFAS Score. The first column lists the parameters calculated from patient examinations, the second and third columns show the correlation coefficients (Pearson’s and Spearman’s rank), and the fourth column presents the significance of values calculated for Pearson’s correlation coefficient using a *t*-test (*p*-value). Results showing a low correlation (above 0.3 or below −0.3) are highlighted. For ‘engagement’, both correlation coefficients indicate a low correlation but with low significance (*p*-value > 0.05; *p* = 0.086). For ‘torsion’, only Spearman’s rank correlation coefficient shows a low correlation, while Pearson’s correlation coefficient is close to the threshold of −0.3 (value = −0.296); significance remains low (*p*-value > 0.05; *p* = 0.111). The remaining values indicate no correlation.

EFAS Correlation			
WBCT Parameter	Pearson	Spearman’s	*p*-Value
Depth	−0.13362	−0.025584	0.4810843
Engagement	**−0.31818**	**0.32157953**	0.0858862
Rotation	0.237927	−0.2483871	0.2047857
Protrusion	−0.21222	0.2189099	0.2595677
Torsion	−0.2961	**0.30945495**	0.1113696
Radiograph Parameter			
Ankle Joint Space	−0.03263	0.05083426	0.8640211
Lateral Clear Space	0.06437	−0.0119021	0.7352403
Medial Clear Space	0.12199	0.02847608	0.5204047
Fibular Overlap	−0.16697	0.2185762	0.3773262
Syndesmosis Width (Mortise)	−0.22042	0.28626251	0.2411493

**Table 5:** Correlations between measured parameters and the FAAM Score. The first column lists the parameters calculated from patient examinations, the second and third columns show the correlation coefficients (Pearson’s and Spearman’s rank), and the fourth column presents the significance of values calculated for Pearson’s correlation coefficient using a *t*-test (*p*-value). Values exceeding 0.3 (or below −0.3) and *p*-values below 0.05 are highlighted. ‘Engagement’ and ‘protrusion’ show low correlation in both correlation coefficients with high significance (*p* < 0.05; values: 0.010 for ‘engagement’ and 0.040 for ‘protrusion’). ‘Syndesmosis width (mortise)’ exhibited a low correlation level using Spearman’s rank correlation coefficient, while Pearson’s correlation coefficient indicated no correlation (−0.181). The significance was low (*p*-value > 0.05; *p* = 0.336).

FAAM Correlation			
WBCT Parameter	Pearson	Spearman’s	*p*-Value
**Depth**	−0.18584	−0.02703	0.324913
Engagement	**−0.45669**	**0.431146**	**0.010847**
Rotation	−0.08174	−0.00634	0.667414
Protrusion	**−0.37582**	**0.339822**	**0.040087**
Torsion	−0.1369	0.174082	0.470262
Radiograph Parameter			
Ankle Joint Space	−0.2398	0.261958	0.201124
Lateral Clear Space	−0.22272	0.107119	0.236158
Medial Clear Space	0.189204	−0.02781	0.316076
Fibular Overlap	0.006599	0.06396	0.972376
Syndesmosis Width (Mortise)	−0.18155	**0.359121**	0.336428

**Table 6:** Correlations between measured parameters and the AOFAS Score. The first column lists the parameters calculated from patient examinations, the second and third columns show the correlation coefficients (Pearson’s and Spearman’s rank), and the fourth column presents the significance of values calculated for Pearson’s correlation coefficient using a *t*-test (*p*-value). Only ‘lateral clear space’ (highlighted) shows a low correlation using Pearson’s correlation coefficient but with low significance (*p*-value = 0.064). The values calculated for ‘engagement’ were close to surpassing 0.3 (or dropping below −0.3), with values of −0.285 for Pearson’s and 0.286 for Spearman’s rank.

AOFAS Correlation			
WBCT Parameter	Pearson	Spearman’s	*p*-Value
Depth	0.028252	−0.18754171	0.882116
Engagement	−0.28543	0.286095662	0.125536
Rotation	0.075188	−0.12625139	0.692729
Protrusion	−0.15533	0.129810901	0.411958
Torsion	−0.21243	0.145272525	0.259103
Radiograph Parameter			
Ankle Joint Space	−0.04738	0.098776418	0.803521
Lateral Clear Space	**−0.34131**	0.066518354	0.064221
Medial Clear Space	0.224759	−0.01145717	0.231766
Fibular Overlap	−0.00817	0.111457175	0.965813
Syndesmosis Width (Mortise)	−0.0484	0.150723026	0.799374

**Table 7:** Correlations between measured parameters and the Olerud–Molander Score. The first column lists the parameters calculated from patient examinations, the second and third columns show the correlation coefficients (Pearson’s and Spearman’s rank), and the fourth column presents the significance of values calculated for Pearson’s correlation coefficient using a *t*-test (*p*-value). Only ‘engagement’ correlated with the Olerud–Molander Score using Pearson’s correlation coefficient (highlighted). Spearman’s correlation coefficient was close to 0.3 (0.298), with a *p*-value slightly above 0.05 (0.066), indicating low significance (*p* > 0.05).

Olerud–Molander Correlation			
WBCT Parameter	Pearson	Spearman’s	*p*-Value
Depth	−0.067861	−0.1917686	0.721431
Engagement	**−0.33867**	0.29833148	0.066456
Rotation	0.0418481	−0.1649611	0.826101
Protrusion	−0.284875	0.1676307	0.126305
Torsion	−0.1364	0.21535039	0.471914
Radiograph Parameter			
Ankle Joint Space	−0.108509	0.11946607	0.567852
Lateral Clear Space	−0.29254	0.20711902	0.115958
Medial Clear Space	0.1494031	0.03982202	0.430262
Fibular Overlap	−0.039698	0.11112347	0.834913
Syndesmosis Width (Mortise)	−0.188373	0.24621802	0.318247

## Data Availability

The database utilized and examined in the course of the present study is accessible to all authors upon reasonable request. To guarantee the confidentiality of the data and to avoid data loss or alteration, the data are restricted to authorized members only. Authorized members are Malik Mateusz, Jakub Kwiatkowski, Artur Gądek, Agnieszka Lechowska-Liszka, and Henryk Liszka. All information about the patients included in our study is saved in our protected server, and database access is possible only through a password that regularly changes. Only the authors have the access key.
